# The Effect of Nutrients on Subjective Accomplishment at Work: Results from a Health Survey and a Single-Arm Dietary Intervention Study

**DOI:** 10.3390/nu16101410

**Published:** 2024-05-08

**Authors:** Takayuki Kawai, Hiroyo Kagami-Katsuyama, Koji Satoh, Takashi Futami, Hiromi Kimoto-Nira, Jun Nishihira, Katsuhisa Tanaka, Takashi Matsumoto, Hajime Shimazaki, Satoshi Yagi, Kohei Sase, Kenji Tanigawa, Mari Maeda-Yamamoto

**Affiliations:** 1 Institute of Food Research, National Agriculture and Food Research Organization, Tsukuba 305-8642, Japan; 2 Department of Medical Management and Informatics, Hokkaido Information University, Ebetsu 069-8585, Japan; 3 FLOWING Co., Ltd., Himeji 679-2162, Japan; 4 NxtQOL Connect Co., Ltd., Kobe 653-0832, Japan; 5 Medical Front Co., Shinjuku-ku 160-0023, Japan; 6 Department of Health and Welfare, Kameyama City Office, Kameyama 519-0164, Japan; 7 Department of Internal Medicine, Kameyama Municipal Medical Center, Kameyama 519-0163, Japan

**Keywords:** subjective accomplishment, daytime sleepiness, Sukoyaka Health Survey, presenteeism, nutrient intake

## Abstract

In Japan, many workers are exposed to chronic stress, sleep deprivation, and nutritional imbalance. They tend still to go to work when ill, leading to decreased work performance and productivity, which has become a major social problem. We conducted a human entry study with the aim of finding a link between these two factors and proposing an optimized diet, believing that a review of diet may lead to an improvement in labor productivity. In this study, we used subjective accomplishment (SA) as a measure of productivity. First, we compared nutrient intake between groups with high and low SA using data from a health survey of 1564 healthy male and female adults. Significant differences were found in the intake of 13 nutrients in males and 15 nutrients in females, including potassium, vitamin A, insoluble fiber, and biotin. Recommended daily intake of these nutrients was determined from survey data. Next, we designed test meals containing sufficient amounts of 17 nutrients and conducted a single-arm intervention study (registration code UMIN000047054) in Kameyama City, Mie Prefecture, Japan. Healthy working adults (males and females aged 20–79 years) were recruited and supplied with test meals, which were eaten once a day 5 days a week for 8 weeks. SA was significantly higher and daytime sleepiness (DS) was significantly lower after lunch on workdays in younger participants (under 60 years) when they ate the test meals as breakfast or lunch. Our results suggest that SA and DS, which change daily, are strongly influenced by the meal eaten before work, and that taking the 17 nutrients may help prevent presenteeism and improve labor productivity.

## 1. Introduction

In recent years, labor productivity decline caused by chronic fatigue, accumulated sleep debt, mental health problems, and physical stress has become a significant problem with personal, social, and economic effects. The GDP per capita in Japan has been the lowest among the seven major industrialized countries since 1994 and has been lower than the average of OECD member countries for more than a decade. This trend has been accelerated by the impact of the new coronavirus pandemic and the depreciation of the yen, and according to a 2023 report, Japan’s labor productivity per capita has stagnated at 31st place out of 38 OECD member countries [1]. Absenteeism and presenteeism are indicators of the impact of health on corporate management. The former is a concept that describes absenteeism, while the latter refers to a state in which a person goes to work feeling unwell or unhealthy but unable to perform at full strength. Presenteeism is considered to be subjectively measurable. The World Health Organization Health and Work Performance Questionnaire (WHO-HPQ) is widely used as a method for evaluating labor productivity decline [2,3]. According to this method, the annual economic loss due to presenteeism in Japan is estimated at approximately 19.3 trillion JPY, or several hundred thousand per person per year [4]. In terms of strategies to improve low labor productivity and presenteeism, it has been reported that making the work environment comfortable, supporting supervisors and coworkers [5], and moderate rest and exercise in the workplace [6] can be helpful. A cross-sectional study reported that irregular eating disrupted circadian rhythms and had a strong impact on presenteeism indirectly through psychological and physical stress reactions and sleep disturbances, and regularization of eating behavior is also considered a useful strategy [7]. Nutrients such as vitamin B_1_, taurine, and caffeine have been included in over-the-counter supplements and drinks, and research on the mechanism of action of their fatigue-relieving effects is ongoing [8,9,10].

Some research reports have considered the effects of a diet consisting of multiple nutrients. By comparing nutrient intake of those who complain of mild decreases in vitality, fatigue, irritability, and somatic issues with those who do not, 17 nutrients associated with these complaints and improvement in presenteeism have been identified [11]. It has been reported that presenteeism can be improved by a 4-week dietary intervention program in which two meals a day are replaced with a COMB meal [12]. However, the nutrients defined in the COMB meal, which aims to provide a completely balanced diet, are a combination of 33 nutrients such as vitamins and minerals that are generally considered important for maintaining good health and are not specifically selected to improve presenteeism. Designing a diet that meets all requirements is complex and difficult. The requirements of COMB meal are very hard to stick to, so it is desirable to select only those nutrients that are important for presenteeism improvement and to minimize the requirements.

Sleep is another parameter that plays a major role in presenteeism. In Japan, there are various factors that cause sleep and body clock disorders, such as shift work, nocturnal lifestyles, and long commutes. According to a 2021 survey report by the Organisation for Economic Co-operation and Development, Japanese people sleep the least among 30 member countries, with an average of 7 h 22 min per night [13]. Compared with other countries, such as China (9 h 1 min), the U.S. (8 h 51 min), and France (8 h 32 min), Japan sleeps considerably less. Persistent short sleep duration and insomnia leads to daytime sleepiness (DS), decreased alertness, and depression, all of which can affect work performance [14].

DS and fatigue caused by elevated blood glucose levels and parasympathetic hyperactivity after eating are physiological phenomena that many people have experienced [15]. Intake of foods high in melatonin, tryptophan, trans fatty acids, and saturated fatty acids tend to make people feel tired after eating [16,17]. There are also reports that avoiding high-carbohydrate diets can suppress postprandial rises in blood glucose levels, and avoiding high-protein diets can maintain adenosine levels in the brain, which may reduce postprandial sleepiness [18,19]. Pearl barley, which is consumed by some Japanese, contains high levels of beta-glucan, a hard digestible dietary fiber, and has been reported to suppress increases in postprandial blood sugar [20].

We hypothesized that physical condition and stress sensitivity, which affect subjective accomplishment (SA) on workdays, may be controllable to some extent by diet. We also hypothesized that differences could be observed in what people with high SA and those with low SA ate. Therefore, the purposes of this study were to identify nutrients associated with SA on workdays from a large survey, and to verify these in an intervention study.

## 2. Materials and Methods

### 2.1. Study 1 (Survey Study)

#### 2.1.1. Design and Population

We analyzed the dataset about SA and dietary nutrient intake obtained from a comprehensive survey to establish an integrated database of food, gut microbiome, and health information (the Sukoyaka Health Survey) [11].

The Sukoyaka Health Survey was conducted on 968 Japanese males and females (age 20–79 years) who worked in Hokkaido and Tokyo, except for patients with serious cerebrovascular disease, heart disease, liver disease, kidney disease, gastrointestinal disease, or infection requiring notification. In the Sukoyaka Health Survey, two surveys were conducted on the same subjects for seven sequential days in summer and winter. The Sukoyaka Health Survey was conducted in the fiscal years of 2019 and 2020 as part of the Strategic Innovation Creation Program (SIP) project. In this study, we analyzed data obtained from subjects who gave written informed consent in the fiscal years of 2019 and 2020 at Hokkaido Information University.

#### 2.1.2. Measurement of SA in the Sukoyaka Health Survey

Participants evaluated SA before lunch and after lunch and scored them separately. The WHO-HPQ [2,3], a presenteeism measurement scale proposed by the World Health Organization, asks questions to ascertain performance position in a group performing similar work, resulting in ranked positions such that the top performer is given a perfect score and the lowest is given a zero. The group of participants in both the Sukoyaka Health Survey and the intervention study had various occupations and lifestyles; therefore, the WHO-HPQ was considered unsuitable. As a result, we asked participants to create a scale tailored to their individual needs, with a score of 0 for the state in which they could not accomplish “what they wanted to do” or “what they had to do” at all, and a score of 10 for the state in which they were able to do so easily and as they wished, and to rate their performance accordingly. Participants recorded SA in a daily questionnaire every day before sleep during the survey period over seven consecutive days in summer and winter. SA obtained in summer and winter from the same participant was analyzed independently.

#### 2.1.3. Estimation of Dietary Nutrient Intakes in the Sukoyaka Health Survey

Participants completed meal record forms (breakfast, lunch, dinner, and snacks) [21] during the study period, measuring food weights on a kitchen scale. In addition, meal photographs were taken whenever possible. A dietitian worked with the participants to minimize missing information by reviewing the contents of the food record questionnaires and food photographs with the participants, and then calculated the daily nutrient intake for each individual participant. Calculations were made according to the Japanese Standard Tables of Food Composition 2015 (7th edition) [22]. Thus, estimated daily intake of nutrients (total energy, water, protein, animal protein, vegetable protein, fat, animal fat, vegetable fat, carbohydrate, ash, sodium, potassium, calcium, magnesium, phosphorus, iron, zinc, copper, vitamin A, retinol, beta-cryptoxanthin, beta-carotene equivalents, vitamin D, vitamin E, vitamin K, vitamin B_1_, vitamin B_2_, niacin, vitamin B_6_, vitamin B_12_, folic acid, pantothenic acid, vitamin C, saturated fatty acids, monounsaturated fatty acids, polyunsaturated fatty acids, cholesterol, total dietary fiber, soluble dietary fiber, insoluble dietary fiber, *n*-3 fatty acids, *n*-6 fatty acids, triacylglycerol equivalents, manganese, iodine, selenium, chromium, molybdenum, and biotin) were obtained.

#### 2.1.4. Analysis of Nutrients Associated with SA

From the Sukoyaka Health Survey, we compared nutrient intake calculated from the dietary record questionnaires of participants with high SA (those who scored an average of seven or more points for SA on workdays during the study period) with those of participants with low SA (those who scored less than seven points) in males and females. We identified nutrients with significantly different intake between the participants with high and low SA levels and included these nutrients in the design of test meals for the intervention study. For improvement in SA, we determined the recommended daily intake of each nutrient to be above the mean intake of the subpopulation with low SA and below the mean intake of the subpopulation with high SA.

#### 2.1.5. Data Analysis in the Sukoyaka Health Survey

Mann–Whitney U tests were used to compare the difference between nutrient intake in participants with high and low SA levels. Male participants were divided into five subgroups according to the number of nutrients consumed above the recommended amount: 0–3, 4–6, 7–9, 10–11, or 12–13. Female participants were also divided into five subgroups according to the number of nutrients consumed above the recommended amount: 0–3, 4–6, 7–9, 10–12, or 13–15. SA on workdays for each group was compared with the 0–3 subgroup. Student’s *t*-tests were performed to compare SA on workdays among nutrient subgroups. All statistical analyses were performed using IBM SPSS Statistics 25. A *p* value < 0.05 was considered statistically significant.

### 2.2. Boxed-Lunch Design with Multiple Nutrients Associated with SA

Ten meals were designed as boxed lunches containing nutrients that were identified as associated with SA on workdays from the survey part of the study.

### 2.3. Study 2 (Intervention Study)

#### 2.3.1. Design and Population

A single-arm intervention study was performed to test the effects of nutrients associated with SA and DS. The participants were recruited from the Kameyama city office in Mie Prefecture (Japan) and were fully informed regarding the content and methods of the study. A lead-in period was observed 10–20 days prior to the start of test meal consumption. The participants then consumed test meals for 8 weeks, with one test meal a day, five test meals a week, and rotated through 10 meals four times. Each participant was interviewed about their health on the day of enrollment and the day after the intervention. Participants reported their meals and physical condition, such as SA and DS before/after lunch very weekday during the lead-in period and the intake period; the meal report contained the time they ate a test meal. Enrollment for the first volunteers started on 7 January 2022, and the study was completed in March 2022. This study was conducted as part of the SIP project.

#### 2.3.2. Participants in the Intervention Study

We recruited healthy Japanese volunteers (aged 30–79 years) who worked in Kameyama city. We enrolled 56 younger volunteers (aged 30–59 years) who worried about metabolic syndrome and 44 older volunteers (aged 60–79). Each participant had their own mobile phone and could use the application to report meal records and physical condition. Participants did not have any food allergies. All participants had sufficient freezing space and a microwave to store and heat test meals, respectively.

#### 2.3.3. Outcomes of the Intervention Study

The primary outcomes were measured with the Brief Job Stress Questionnaire (BJSQ) [23], which records vigor, irritability, fatigue, depression, physical complaints, psychological and physical stress responses, and SA on workdays.

#### 2.3.4. Measurement of Psychosomatic Disorders in the Intervention Study

The BJSQ is a 29-item questionnaire querying psychological and physical stress reactions. We modified the target period of the questionnaire from a month to a week. Participants answered the 29-item questionnaire in the application on their mobile phone once a week. This questionnaire was designed by the Stress Measurement Study Group of the Study Group on the Prevention of Work-Related Diseases of the Ministry of Health, Labour, and Welfare [23].

#### 2.3.5. Measurement of SA and DS in the Intervention Study

Participants evaluated SA before lunch (8:00–12:00) and after lunch (14:00–18:00) and scored them separately. A score of 0 was given when no progress was made, and a score of 10 was given when complete progress was made. They also evaluated DS before lunch (8:00–12:00) and after lunch (14:00–18:00) as none, weak, or strong. Participants recorded these results in the application on their mobile phone every weekday. They also recorded whether it was a workday or a rest day.

#### 2.3.6. Data Analysis in the Intervention Study

The BJSQ job stress metrics of vigor, irritability, fatigue, anxiety, and somatic symptoms were obtained for 9 weeks. The data were divided into three periods: an early period (weeks 0–2), a middle period (weeks 3–5), and a final period (weeks 6–8). Paired *t*-tests were used to measure the statistical significance of changes in BJSQ-assessed job stress during these periods of the intervention study.

An SA score was averaged across the test period for each subject and was determined separately for workdays and rest days. A DS score was calculated by dividing the number of times a participant recorded feeling sleepy by the total number of days recorded for each participant. The DS score was determined separately for workdays and rest days. Paired *t*-tests were used to compare SA and DS scores before and after lunch. Student’s *t*-tests were used to compare SA and DS scores between age groups.

On workdays, SA and DS before and after lunch were compared on days when the test meal was eaten as breakfast or lunch with days when a test meal was not consumed using a paired *t*-test.

All statistical analyses were performed using IBM SPSS Statistics 25. A *p* value < 0.05 was considered statistically significant.

### 2.4. Ethics

The intervention study and the Sukoyaka Health Survey were conducted following the ethical principles of the Declaration of Helsinki (revised by the World Medical Association Fortareza General Assembly in October 2013) and in compliance with the Ethical Guidelines for Medical Research for Persons (revised by the Ministry of Education, Culture, Sports, Science, and Technology and the Ministry of Health, Labour, and Welfare on 28 February 2017). We obtained written informed consent from all subjects. The FY2021 SIP2 NARO Style Plus Lunch Box Implementation Experiment Ethics Committee reviewed and approved the feasibility and the ethical and scientific validity of the intervention study (approval date 6 January 2022; approval number 2021-03). The Bioethics Committee of Hokkaido Information University reviewed and approved the feasibility and the ethical and scientific validity of the Sukoyaka Health Survey (approval date 22 April 2019; approval number 2019-04).

## 3. Results

### 3.1. Study 1 (Survey Study)

#### 3.1.1. Participant Selection in the Sukoyaka Health Survey

**Figure 1:**
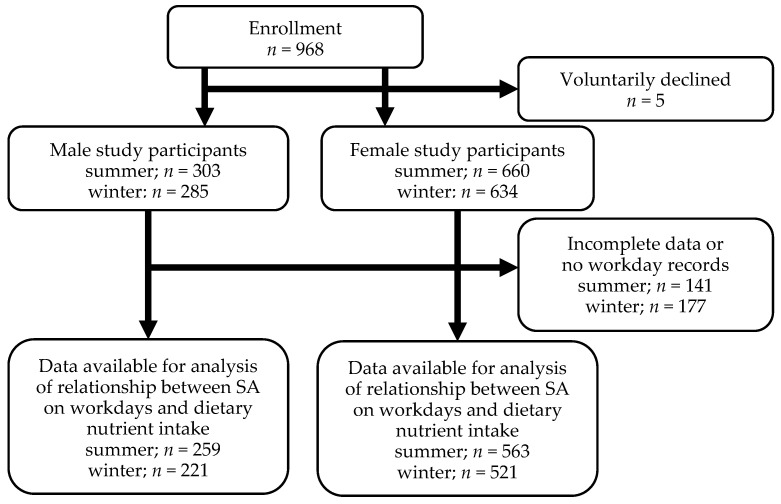
Flowchart of participant selection and participation in the Sukoyaka Health Survey. *n*: number of participants; SA: subjective accomplishment.

Figure 1 shows the flowchart of participant selection and participation in the survey study. First, 968 participants were enrolled. Excluding those who voluntarily withdrew, there were 303 males and 660 females in the summer session, and 285 males and 634 females in the winter session The number of participants whose questionnaires were complete and who had at least one workday during the survey period was 259 males and 563 females in the summer session, and 221 males and 521 females in the winter session. The gender composition was 3:7 for male-to-female participants. The majority of the age group was from 40 to 59 years old. Characteristics of the data-available participants in the survey study are shown in Table 1. In total, we included 1564 participants in the analysis as the “working participant population.”

#### 3.1.2. SA in the Sukoyaka Health Survey

**Figure 2:**
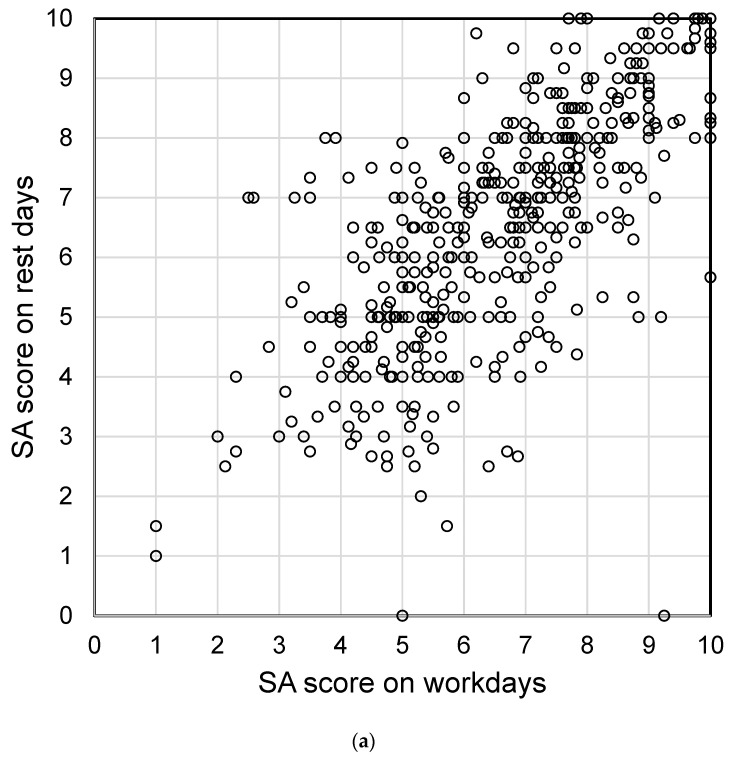

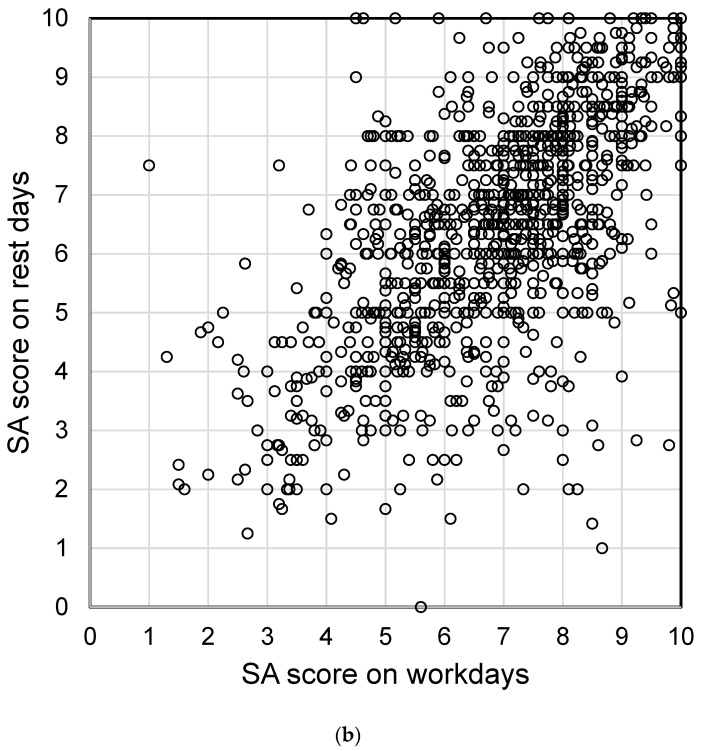
Comparison of subjective accomplishment (SA) scores between workdays and rest days in the Sukoyaka Health Survey. (**a**) Male participants who had both workday and rest day data during the survey period (*n* = 474, *r* = 0.71, *p* = 0.280). (**b**) Female participants who had both workday and rest day data during the survey period (*n* = 1058, *r* = 0.63, *p* < 0.001).

**Figure 3:**
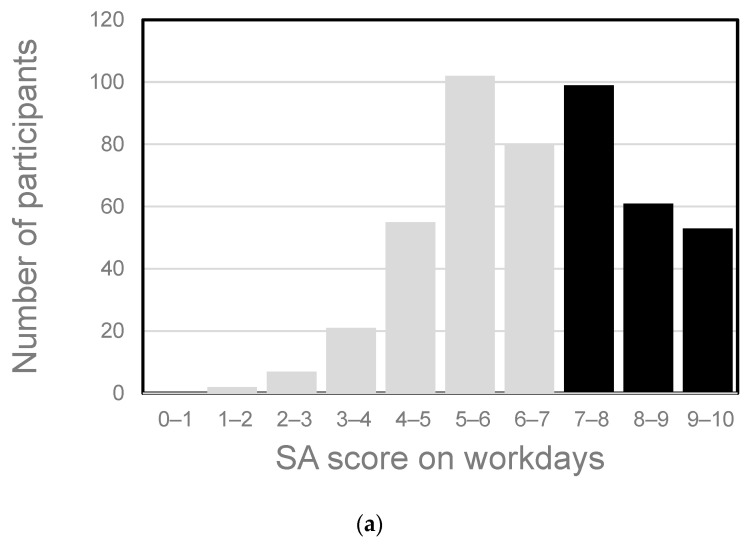

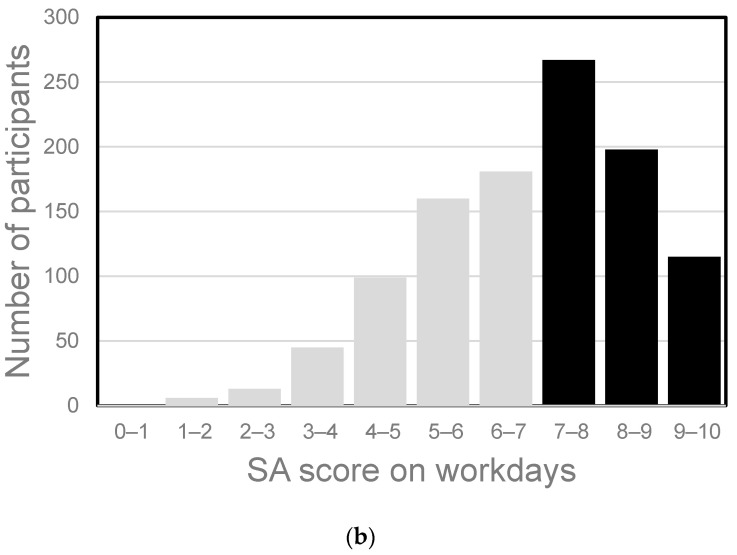
Distribution of subjective accomplishment (SA) scores in the Sukoyaka Health Survey. (**a**) Male participants (*n* = 480), (**b**) female participants (*n* = 1084). SA score on workdays was averaged individually. The grey bars show the subpopulation with low SA, the black bars show the subpopulation with high SA.

The SA score on workdays was the average of workday scores, and that on rest days was the average of rest day scores. Figure 2 shows the correlation between SA on workdays and rest days for the 272 male and 590 female participants who had data for work and rest days during the same survey period. Although SA on workdays was generally correlated with SA on rest days (correlation coefficient (*r*): male 0.71, female 0.63), there were some participants whose SA on workdays diverged from that on rest days. Statistical analysis showed significant differences between SA on workdays and rest days in females. The distribution of SA on workdays is shown in Figure 3. The mean score was 6.6 for males and 6.8 for females, and the median score was 6.6 for males and 7.0 for females. Based on these results, we considered a score of seven or higher to denote high SA and a score of less than seven to denote low SA.

#### 3.1.3. Survey for the Nutrients Associated with SA on Workdays

In the Sukoyaka Health Survey, we compared nutrient intake calculated from the dietary records of participants with high SA who scored an average of seven or more points for SA on workdays during the study period and those of participants with low SA who scored less than seven points in males and females. Significant differences were observed in the intake level of 19 nutrients between participants with high and low SA. Table 2 shows the intake of each group for the 19 nutrients, excluding water.

Male participants with high SA consumed significantly higher levels of water, potassium, magnesium, copper, vitamin A, beta-cryptoxanthin, beta-carotene, vitamin D, vitamin E, vitamin B_12_, folate, pantothenic acid, vitamin C, total dietary fiber, insoluble dietary fiber, *n*-3 fatty acids, and biotin than those with low SA (all *p* < 0.05). Female participants with high SA consumed significantly higher levels of water, animal fat, ash, potassium, magnesium, phosphorus, iron, vitamin A, retinol, vitamin D, vitamin B_12_, folic acid, vitamin C, cholesterol, insoluble dietary fiber, selenium, and biotin than those with low SA (all *p* < 0.05). For both males and females, there was no relationship between SA on workdays and total energy intake, carbohydrate intake, or fat intake.

**Figure 4:**
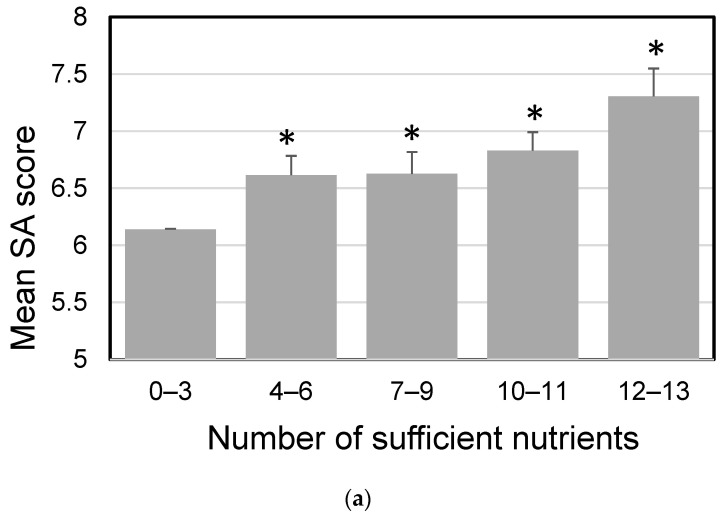

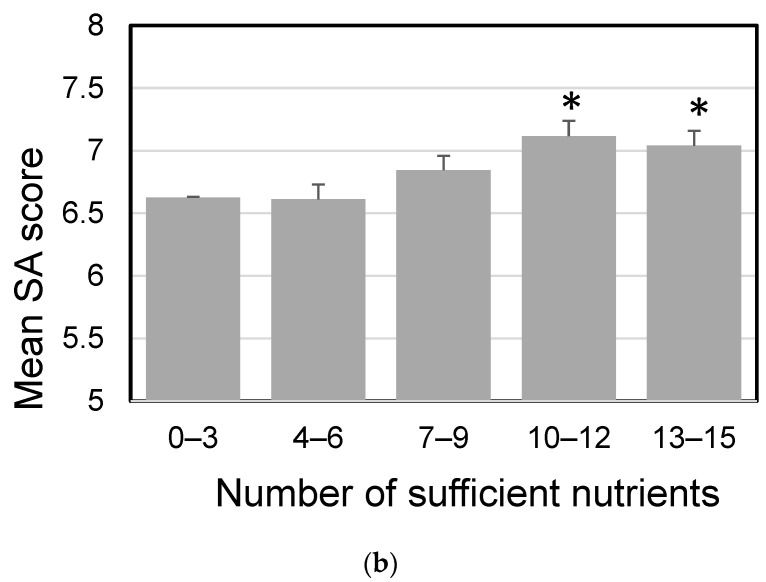
Mean subjective accomplishment (SA) on workdays in each subgroup. (**a**) Male participants (*n* = 480), (**b**) female participants (*n* = 1084). Bars represent means ± standard error. Asterisks show significant differences compared with the 0–3 group according to Mann–Whitney U tests (*p* < 0.05).

Nutrients with significantly different intake between participants with high and low SA were used to design the test meals in the intervention study. To improve SA, we set the recommended daily intake of the nutrients to be above the mean intake of the subpopulation with low SA and below the mean intake of the subpopulation with high SA. The recommended amount of nutrients is shown in Table 2. SA on workdays in the five subgroups divided by the number of nutrients consumed in sufficient amounts is shown in Figure 4. Compared with consuming zero to three nutrients at sufficient levels, groups consuming four or more nutrients at sufficient levels in males, and ten or more nutrients at sufficient levels in females, had significantly higher SA scores (all *p* < 0.05).

### 3.2. Boxed-Lunch Design with Multiple Nutrients Associated with SA

Ten meals were designed to contain at least one third of the recommended daily intake of the 19 nutrients associated with high SA on workdays. The calculated content in the meals exceeded one third of the male recommended amount for 11 nutrients: potassium, magnesium, copper, vitamin A, vitamin E, vitamin B_12_, folic acid, pantothenic acid, vitamin C, insoluble dietary fiber, and *n*-3 fatty acids. It also exceeded one third of the female recommended amount for 12 nutrients: potassium, magnesium, phosphorus, iron, vitamin A, vitamin D, vitamin B_12_, folic acid, vitamin C, insoluble dietary fiber, selenium, and biotin. The calculated content of the major nutrients in the 10 meals is shown in Table 3. To ensure insoluble fiber content, in all meals, pearl barley mixed with rice was offered. Although some nutrient contents may be reduced due to cooking and processing, the nutrient intake in the survey study was estimated based on the content of ingredients; therefore, the design of meals was similarly based on food ingredients.

### 3.3. Study 2 (Intervention Study)

#### 3.3.1. Participants in the Intervention Study

**Figure 5:**
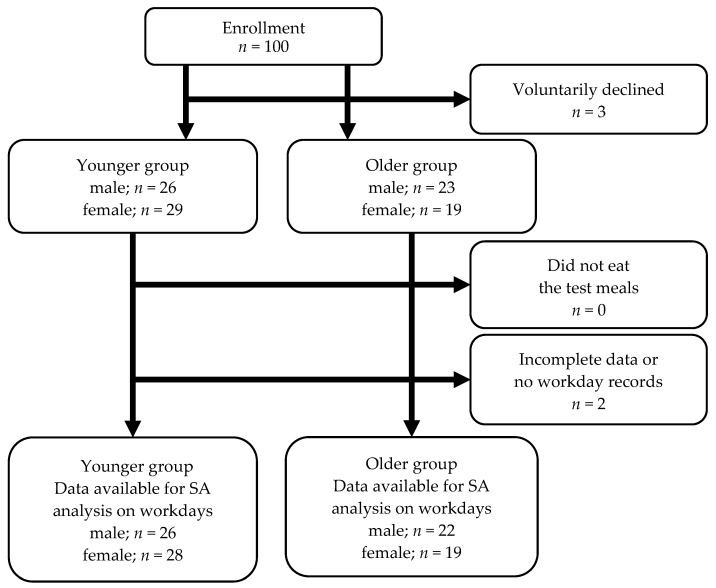
Flowchart of participant selection and participation in the intervention study. The younger group consisted of participants under 60 years old and the older group consisted of participants 60 years old and older. n: number of participants; SA: subjective accomplishment.

Figure 5 shows a detailed flowchart of the population selected for the intervention study. First, 100 participants were enrolled. The younger group consisted of participants under 60 years old, while the older group consisted of participants 60 years old and older. Excluding those who voluntarily withdrew, there were 49 males and 48 females. The participants whose questionnaires were complete and who had at least one workday during the survey period numbered 26 males and 28 females in the younger group and 22 males and 19 females in the older group. The gender composition was 1:1 for male-to-female participants. Characteristics of the participants in this study are shown in Table 4. Ultimately, we included 95 participants in the analysis as the “working participant population”.

#### 3.3.2. Psychosomatic Disorders in the Intervention Study

**Figure 6:**
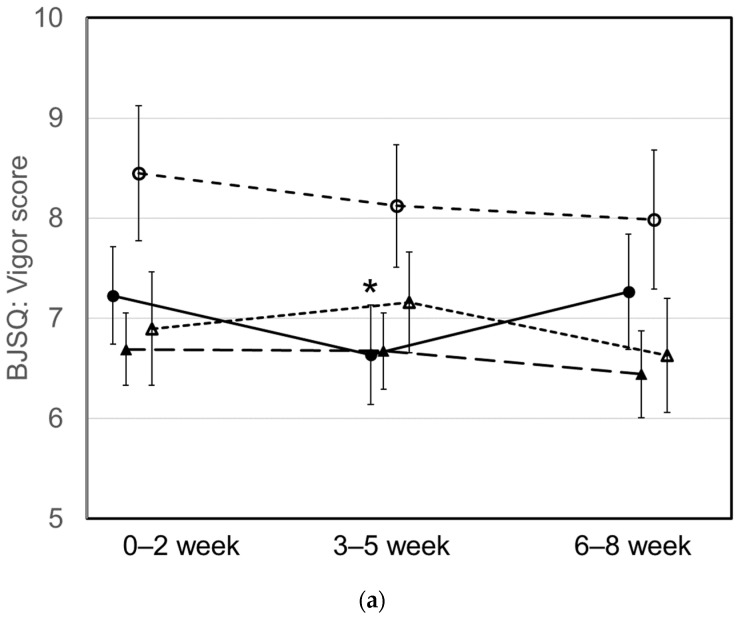

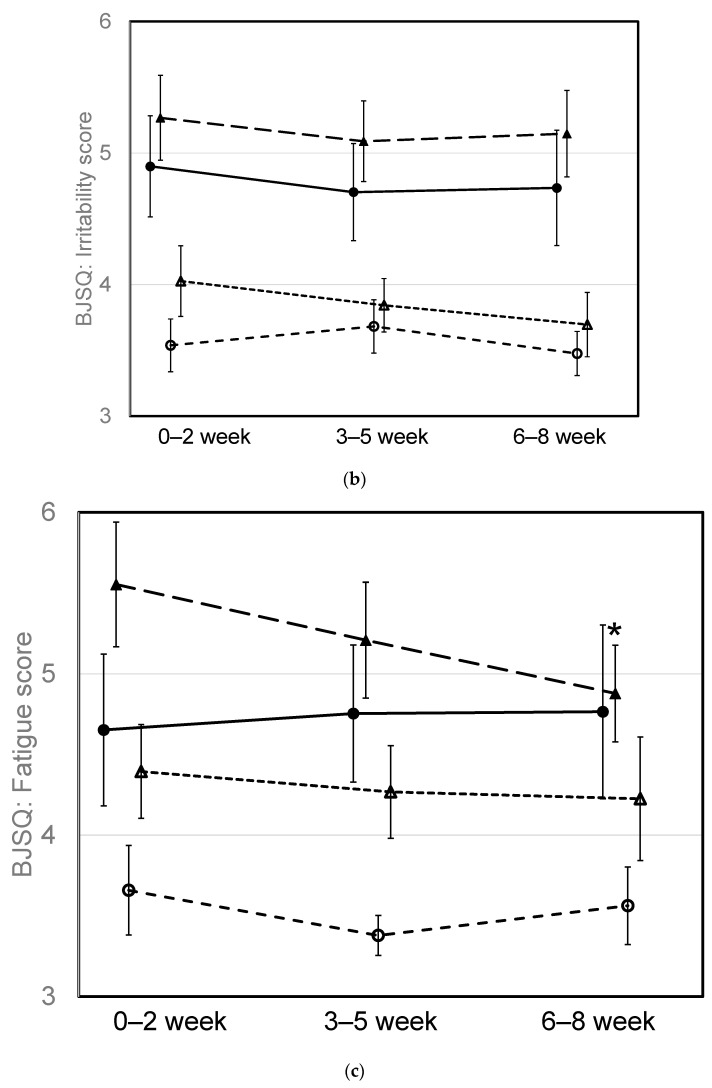

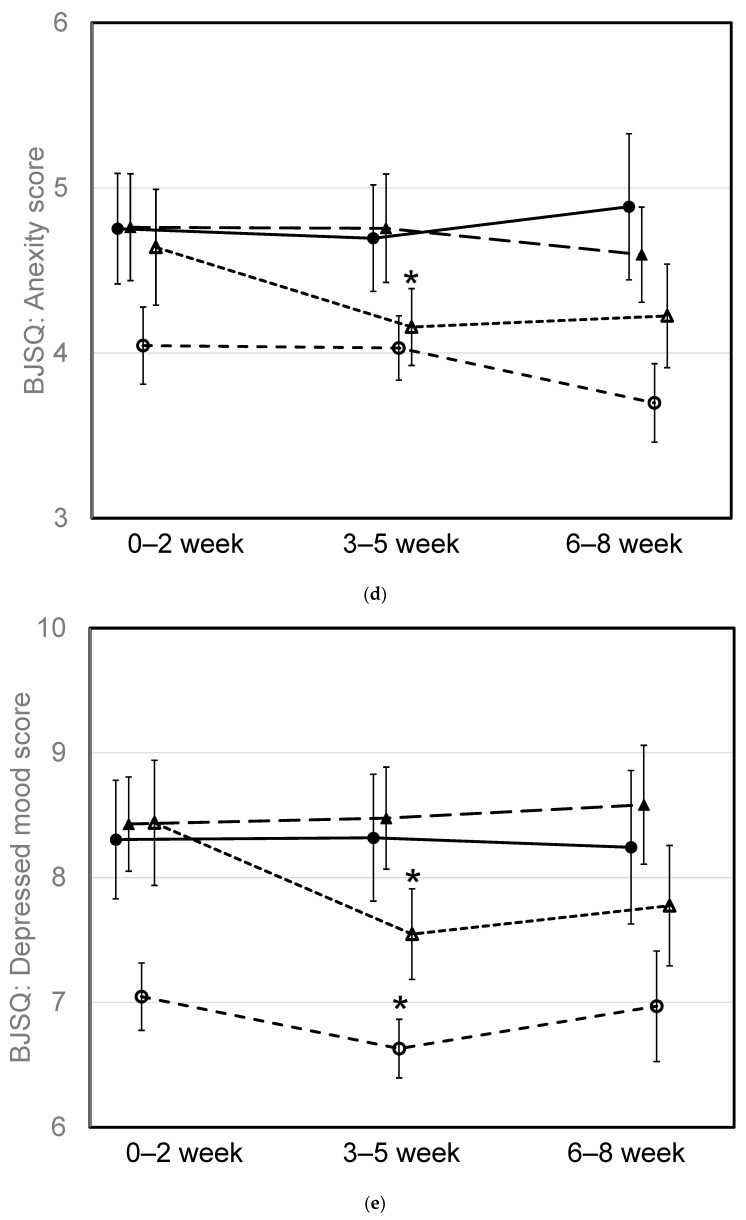

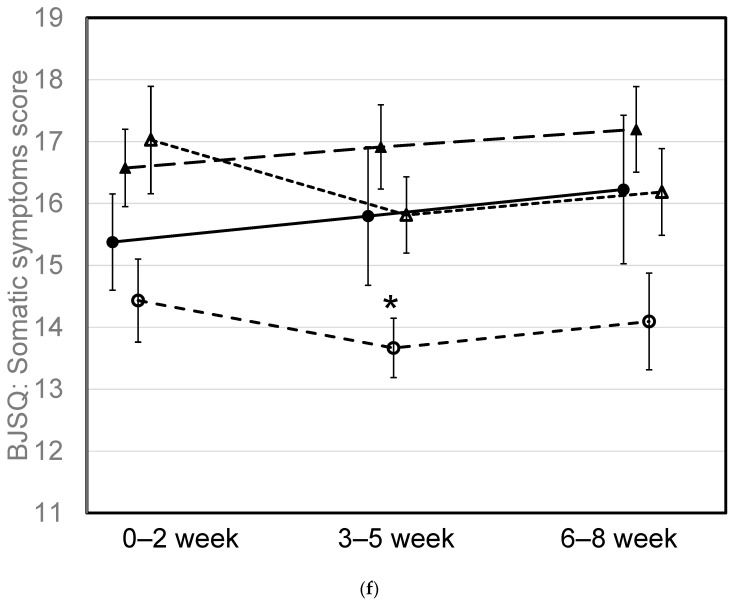
Changes in job stress measured by the Brief Job Stress Questionnaire (BJSQ) during the intervention period. Closed circles show average scores of younger males, closed triangles of younger females, open circles of older males, and open triangles of older females. (**a**) Vigor score, (**b**) irritability score, (**c**) fatigue score, (**d**) anxiety score, (**e**) depressed mood score, (**f**) somatic symptoms score. Data points represent means ± standard error. Asterisks show significant differences compared with the 0- to 2-week period according to paired *t*-tests (*p* < 0.05).

During the 8-week intervention study, reports on job stress were collected weekly. Stress scores were compared for 3 weeks from 1 week before to 2 weeks after the start of intervention (early period), for 3 weeks from the third to the fifth week of the intervention period (middle period), and for 3 weeks from the sixth to the eighth week of the intervention period (final period; Figure 6). Compared with the early period, there was a significant decrease in anxiety and depression in older females and a significant decrease in depression and somatic complaints in older males in the middle period (all *p* < 0.05). There was also a significant decrease in fatigue in younger females in the final period (*p* < 0.05). A significant decrease in vitality in younger males observed in the middle period of the intervention compared with the early period (*p* < 0.05) was no longer evident in the final period (*p* > 0.05).

#### 3.3.3. SA in the Intervention Study

**Figure 7:**
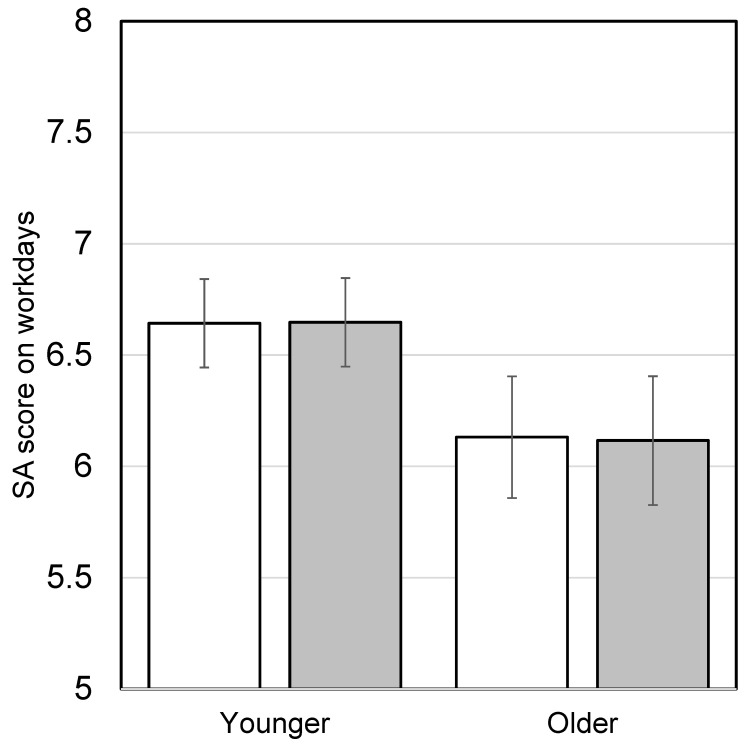
Subjective accomplishment (SA) before and after lunch on workdays in the intervention study. The younger group consisted of participants under 60 years old, and the older group consisted of participants 60 years old and older. Bars represent means ± standard error. White boxes show SA scores before lunch and gray boxes show SA scores after lunch (younger, *n* = 54; older, *n* = 41). There were no significant differences between scores before and after lunch according to paired *t*-tests (*p* < 0.05).

Participants evaluated SA before lunch (8:00–12:00) and after lunch (14:00–18:00) and scored them separately. A score of 0 was given when no progress was made, and a score of 10 was given when complete progress was made. SA scores before and after lunch on workdays are shown in Figure 7. No significant differences were observed between SA before lunch and after lunch in either the younger or older group.

#### 3.3.4. DS in the Intervention Study

**Figure 8:**
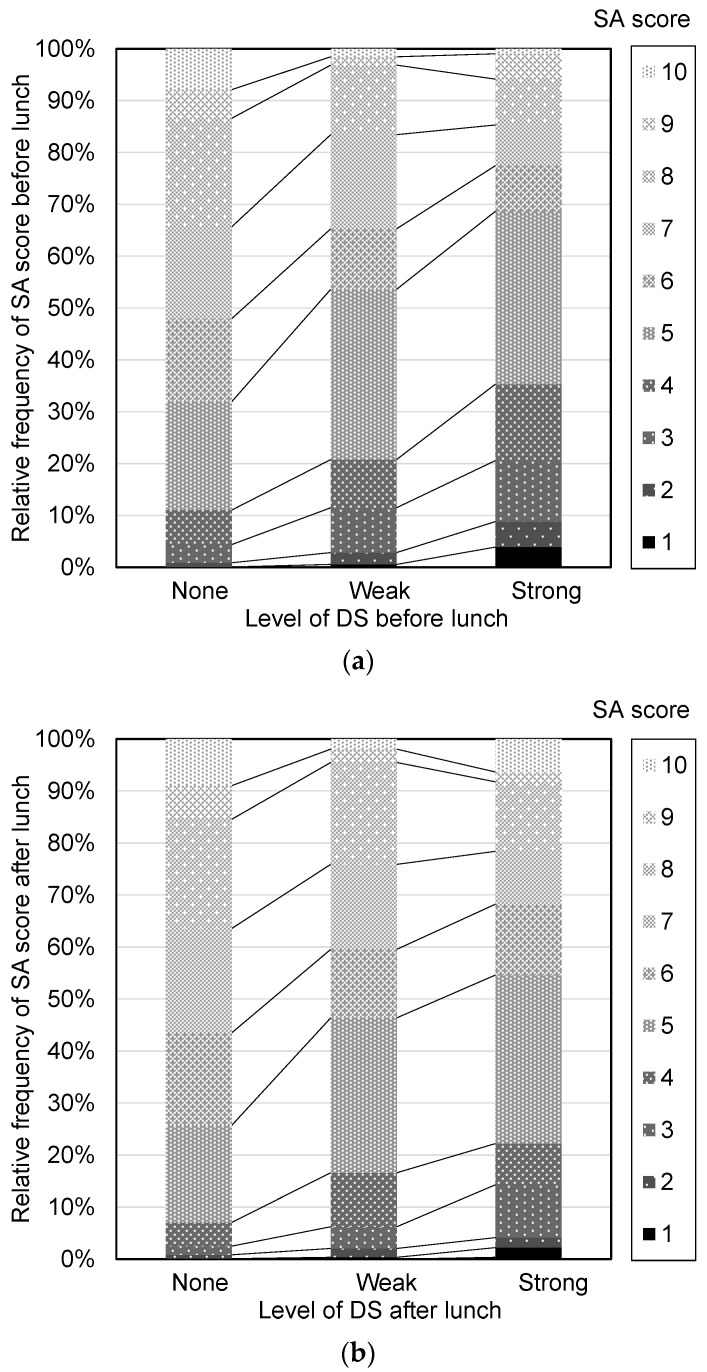
Relationship between daily subjective accomplishment (SA) on workdays and daytime sleepiness (DS) in the intervention study. (**a**) SA scores expressed as relative frequencies (%) before lunch on workdays (no DS, *n* = 3376 days; weak DS, *n* = 881 days; strong DS, *n* = 102 days). (**b**) SA scores expressed as relative frequencies (%) after lunch on workdays (no DS, *n* = 2517 days; weak DS, *n* = 1527 days; strong DS, *n* = 315 days). The participants included had at least one workday recorded during the intervention period.

**Figure 9:**
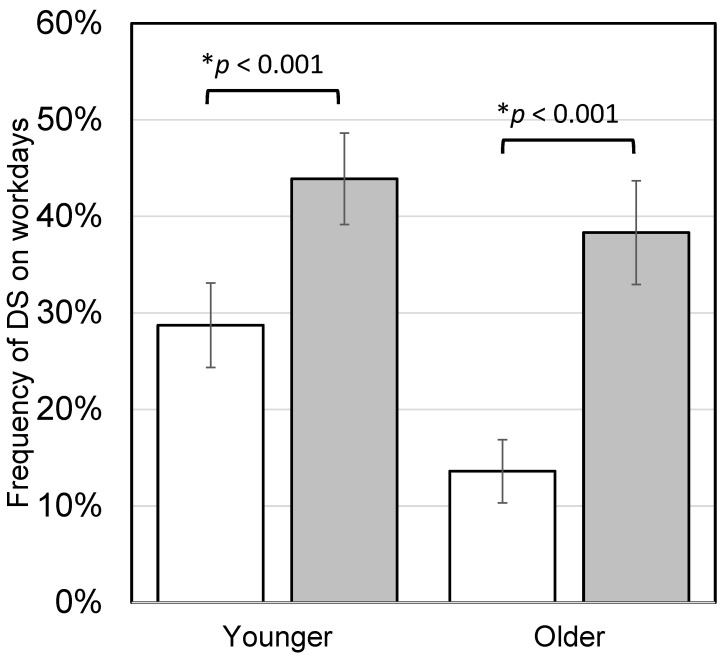
Daytime sleepiness (DS) before and after lunch on workdays in the intervention study. Frequency of DS was calculated individually as the percentage of workdays in which sleepiness occurred during the day out of all workdays, divided into before and after lunch. The younger group consisted of participants under 60 years old, and the older group consisted of participants 60 years old and older. Bars represent means ± standard error. White boxes show the frequency of DS before lunch and gray boxes show the frequency of DS after lunch (younger, *n* = 54; older, *n* = 41). Asterisks show significant differences according to paired *t*-tests.

Participants evaluated DS before lunch (8:00–12:00) and after lunch (14:00–18:00) separately as none, weak, or strong. Figure 8 shows the relationship between SA and DS in the intervention study. Lower SA was associated with higher DS both before and after lunch. The frequency of DS on workdays of subpopulations is shown in Figure 9. The frequency of DS after lunch was significantly higher than before lunch in both younger and older participants (both *p* < 0.001).

#### 3.3.5. Effect of Test Meals on SA and DS

**Figure 10:**
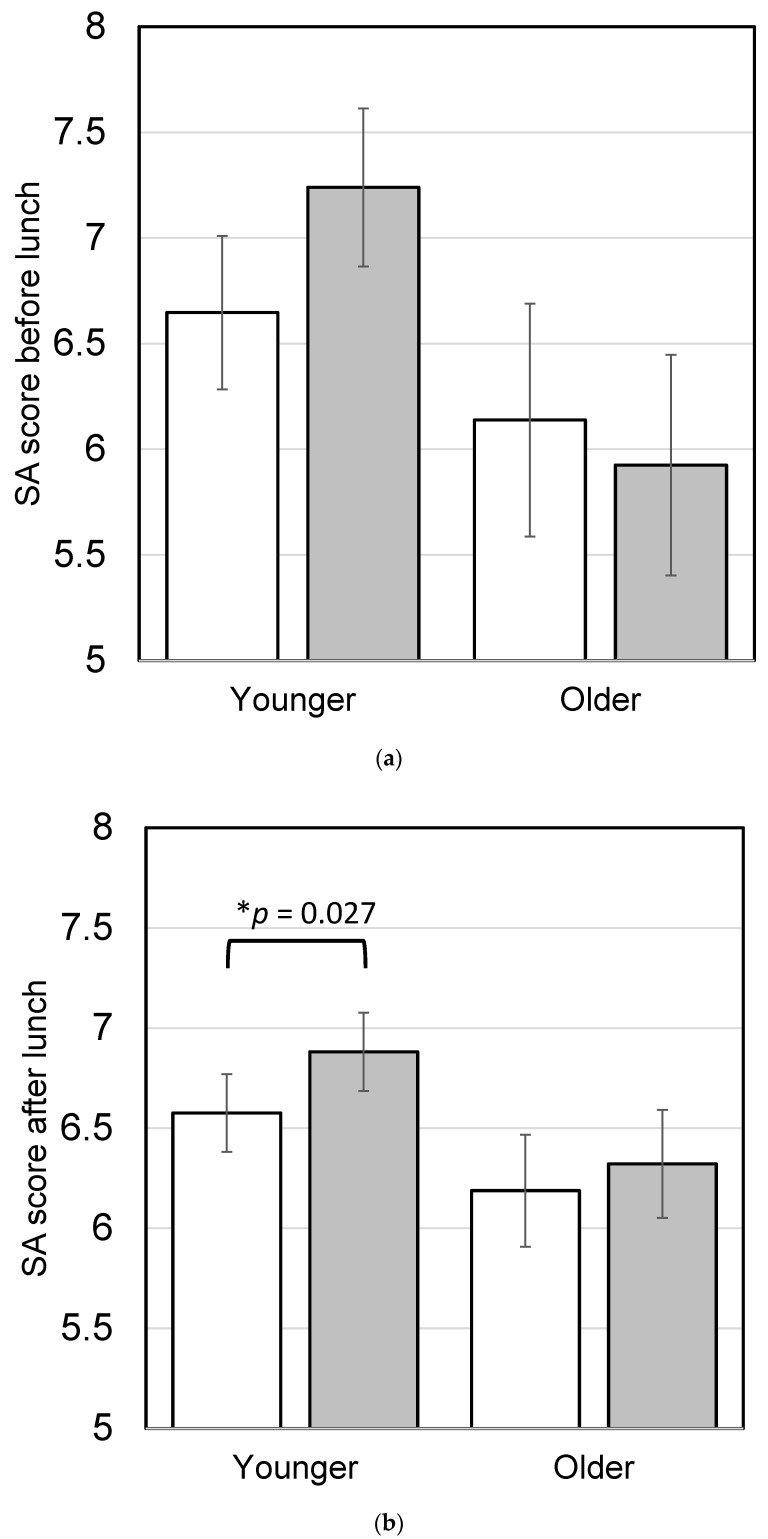
Effect of the test meals on subjective accomplishment (SA) in the intervention study. The younger group consisted of participants under 60 years old, and the older group consisted of participants 60 years old and older. (**a**) SA scores before lunch on workdays. White boxes show the days when participants did not eat a test meal as breakfast (younger, *n* = 54; older, *n* = 41), gray boxes show the days participants ate a test meal as breakfast (younger, *n* = 16; older, *n* = 10). (**b**) SA scores after lunch on workdays. White boxes show the days when participants did not eat a test meal as either breakfast or lunch (younger, *n* = 54; older, *n* = 41), gray boxes show the days participants ate a test meal as breakfast or lunch (younger, *n* = 41; older, *n* = 41). Bars represent means ± standard error. Asterisks show significant differences according to paired *t*-tests.

**Figure 11:**
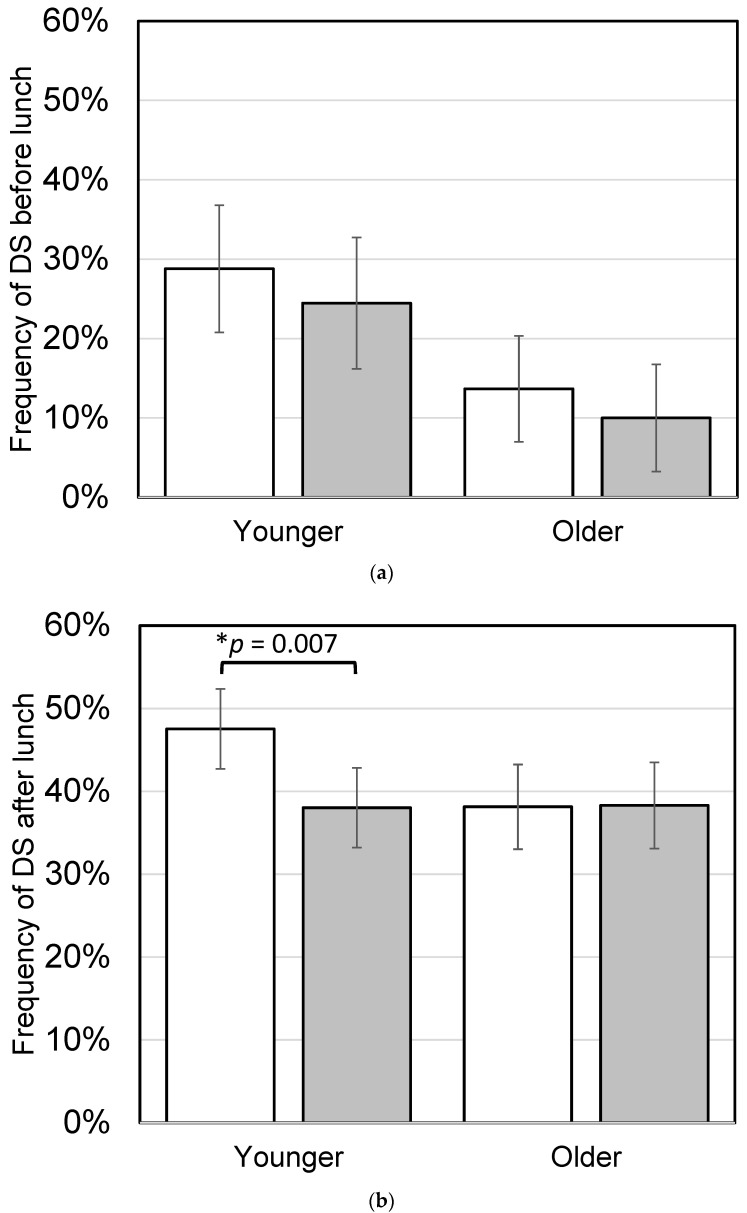
Effect of the test meals on daytime sleepiness (DS) in the intervention study. Frequency of DS was calculated individually as the percentage of workdays in which sleepiness occurred during the day out of all workdays, divided into before and after lunch. The younger group consisted of participants under 60 years old, and the older group consisted of participants 60 years old and older. (**a**) DS before lunch on workdays. White boxes show the days when participants did not eat a test meal as breakfast (younger, *n* = 54; older, *n* = 41), gray boxes show the days participants ate a test meal as breakfast (younger, *n* = 16; older, *n* = 10). (**b**) DS after lunch on workdays. White boxes show the days when participants did not eat a test meal as either breakfast or lunch (younger, *n* = 54; older, *n* = 41), gray boxes show the days participants ate a test meal as breakfast or lunch (younger, *n* = 41; older, *n* = 41). Bars represent means ± standard error. Asterisks show significant differences according to paired *t*-tests.

During the intervention study, participants ate 40 intervention test meals, either as breakfast, lunch, or dinner. To test the effect of mealtime on SA, workdays were divided into four categories: a day in which the test meal was consumed as breakfast, lunch, or dinner, or a day in which no test meal was consumed. Few participants ate the test meal as breakfast, and no significant effect of the test meal eaten as breakfast was observed on SA before lunch (Figure 10a). Younger participants who ate the test meal as breakfast or lunch had significantly higher SA after lunch on workdays (*p* = 0.027; Figure 10b). There was no significant effect of test meals eaten as breakfast on DS before lunch (Figure 11a), but younger participants who ate the test meal as breakfast or lunch had significantly lower DS after lunch on workdays (*p* = 0.007; Figure 11b). Analyzing each of the 10 meals separately showed that all meals were equally effective in improving SA.

## 4. Discussion

The combination of survey and intervention studies revealed several insights into the relationship between nutrient intake and productivity. We hypothesized that labor productivity would fluctuate daily depending on the previous night’s sleep status, nutritional imbalances, and work-related stress, so we created a questionnaire that subjects could easily complete. During the study period, participants recorded subjective accomplishment (SA) before and after lunch and daytime sleepiness (DS) before and after lunch daily. The questionnaire was similar to the Single-Item Presenteeism Question (SPQ) [24], released in 2021 by the Future Vision Research Center of the University of Tokyo, but it was not adopted because it is a questionnaire that is supposed to assess work performance over the past four weeks. Other methods include the WHO-HPQ [2,3], which is suitable for presenteeism comparisons and evaluation in similar occupational groups, and the WLQ [25], which is suitable for looking for causes of presenteeism, but neither of these methods was deemed suitable for daily responses and was not adopted.

The dietary records obtained from the Sukoyaka Health Survey were analyzed to identify nutrients that are candidates for improving SA, and 13 ingredients were found for males and 15 ingredients were found for females (Table 2). However, not all of these nutrients are required in sufficient amounts to improve SA, and some combinations of nutrients are expected to significantly improve SA (Figure 4). Because males and females have different caloric requirements, their nutrient intake also differs. In designing the test meals for the intervention study, it was difficult to personalize the amounts by gender and weight, so test diets were produced that contained amounts that would be sufficient for both males and females. Females with higher SA had higher intake of animal fat and ash derived from sodium, but when those components were increased, possible adverse reactions such as increased blood LDL and cardiovascular disease can occur, so we decided to ignore them. Estimated nutrient intake was calculated separately for males and females, and the higher of the nutrients common to both sexes was used in the design of the test meals. In sum, 16 of the 17 ingredients employed in our test meal, excluding cholesterol, were among the 33 ingredients defined in the COMB meal, which has been suggested to be effective in improving presenteeism [12]. However, there were no reports of direct effects of nutrients alone on improving labor productivity, and the study was also consistent in that the sufficiency of multiple nutrients had an effect. Total calories and total glucose were higher in our test meal than in the COMB meal program. Only our test meal contained barley, which is high in beta-glucan, but we did not measure postprandial blood glucose levels in either study, so we could not consider the effect of blood glucose changes on labor productivity.

The Sukoyaka Health Survey identified and reported components associated with minor health complaints, such as decreases in vitality, fatigue, irritability, and somatic issues [11]. The 10 nutrients that overlapped with candidates for SA improvement were potassium, magnesium, phosphorous, iron, copper, vitamin A, folic acid, pantothenic acid, insoluble dietary fiber, and biotin. These may contribute to improvement in SA through their stress-relieving effects.

In our intervention study, we did not use supplements to increase nutrient content, but relied on the nutrient content of the whole foods contained within meals. Younger participants, under 60 years old, who ate the test meal as breakfast or lunch had significantly higher SA after lunch on workdays. They also had significantly lower DS after lunch on workdays, although it was not possible to identify the ingredients or foodstuffs that contributed to changes in SA and DS in this study. Analyzing the 10 meals separately showed that all meals were effective in improving SA and there was no difference between the meals. As eight components—potassium, magnesium, phosphorus, vitamin A, vitamin E, vitamin C, folic acid, and insoluble fiber—were common to every meal, they may have made a particular contribution to improving SA and suppressing DS (Table 3). However, there were also likely additive and synergistic effects of other nutrients in the meals that cannot be easily identified.

The intervention study showed the relationship between SA and DS and the possibility that avoidance of DS improved SA. We expected that consumption of test meals would have a cumulative effect, but there was no significant difference in SA and DS on days when a test meal was not chosen between the early and final periods of the intervention. This suggests that transient sleepiness may be prevented by some diets, but the effect may not be maintained until the next day. It also suggests that a healthy daily diet is important to maintain good productivity. Results from the intervention also suggest that continued consumption of test meals was somewhat effective in reducing occupational stress, such as fatigue, anxiety, depression, and somatic complaints, although changes were limited and specific to certain age and sex groups.

The survey study was not conducted across Japan, but rather a portion of Hokkaido and a portion of Tokyo, where data could be obtained earlier. Hokkaido and Tokyo are 820 km apart and 8 degrees in latitude. The intervention test was conducted in Kameyama City, more than 300 km away from the two cities. The 17 candidate nutrients would have potential for generalization in Japan. Similar studies have been conducted in other areas of Japan, and the addition of such data would further narrow down the candidate ingredients that are effective in improving SA, although detailed data were not available at the time of writing. In the future, similar studies with some nutrients omitted would help to identify the most effective nutrient candidates and lead to less complicated menus. The information on nutrients related to improving SA may be useful to provide meals to employees at companies that engage in health management.

Both the survey and intervention studies targeted healthy adults. A similar trial is currently being designed for high school students under the age of 20. It is expected that improved SA and decreased DS during school classes will lead to increased learning efficiency. When conducting a comparative study with a placebo meal, the test meal will be based on the diet eaten by those with high SA, and the placebo meal will be based on the diet eaten by those with low SA. There is strong resistance to giving placebo meals to high school students, even for a certain period. In addition, it is difficult to create a test meal and a placebo meal with similar visuals. We must develop a study design in which no one complains.

There were four limitations to this study. First, the timing of eating the boxed lunch was not specified in this intervention study because we expected that eating the boxed lunch continuously would improve the constitution such that high SA would be seen consistently. Therefore, the time between the meal and the SA assessment was not consistent, and the effect size of the boxed lunch could not be represented accurately. Second, we did not examine the nutrients in the regular meals of the participants, although the nutrients in the test meals were well determined. Therefore, it is not possible to consider which of the 17 components were important. In the future, a controlled trial using a placebo meal will be essential. Thirdly, only 100 subjects in total were collected for this intervention study, so the statistical population was too small to analyze young males and young females separately. The study had to be divided into only younger and older participants. In future studies, the study must be designed to ensure enough participants to allow for a sufficient population to be divided into two groups, one on the placebo and the other on the test meal. Finally, the SA assessment used in this study was new to all subjects. There is a risk that the interpretation of the results may deviate from the original reality due to overestimation, underestimation, or unstable evaluation by the respondents. To capture statistically correct changes in SA, it is necessary to unify the evaluation scale.

## 5. Conclusions

This study showed that 17 nutrients, closely related to high and low SA from independently conducted survey studies, could be useful in designing meals to improve workday SA in healthy adults. The intervention study replacing one meal a day containing around one third of the recommended amount of candidate nutrients showed that intake of the meal before work significantly improved SA and DS in healthy adults under 60 years old. This result suggests that SA and DS, which change daily, are strongly influenced by the meal eaten before work, and that taking the 17 nutrients may help prevent presenteeism and improve labor productivity. It also indicates that it may be useful to provide lunch at companies that engage in health management.

## Figures and Tables

**Table 1:** Characteristics of participants in the Sukoyaka Health Survey.

Season	Age Groups	Male		Female	
		*n*	%	*n*	%
summer	20–29	17	2.1	35	4.3
	30–39	43	5.2	85	10.3
	40–49	70	8.5	169	20.6
	50–59	70	8.5	194	23.6
	60–69	49	6.0	64	7.8
	≥70	10	1.2	16	1.9
	Total	259	–	563	–
winter	20–29	12	1.6	27	3.6
	30–39	34	4.6	76	10.2
	40–49	59	8.0	149	20.1
	50–59	58	7.8	187	25.2
	60–69	45	6.1	67	9.0
	≥70	13	1.8	15	2.0
	Total	221	–	521	–

These participants had both nutritional information and questionnaires for at least one workday during the survey period. n: number of participants. %: percentage among total participants in each season.

**Table 2:** Intakes of 19 nutrients in males and females stratified by subjective accomplishment.

	Male				Female			
	Subjective Accomplishment				Subjective Accomplishment			
Nutrients	Low	High	*p*	Cut-Off	Low	High	*p*	Cut-Off
Animal fat (g)	35.5	36.2	0.488	–	29.2	30.1	0.032	>30
Ash (g)	18.5	19.4	0.077	–	16.3	16.9	0.011	>16.5
K (mg)	2504	2703	0.007	>2600	2329	2444	0.009	>2400
Mg (mg)	283	302	0.037	>290	250	263	0.010	>260
P (mg)	1162	1222	0.060	–	1020	1054	0.029	>1030
Fe (mg)	8.40	8.87	0.127	–	7.48	7.85	0.008	>7.5
Cu (mg)	1.23	1.30	0.049	>1.3	1.07	1.09	0.302	–
Vitamin A (μg)	410	463	0.009	>420	424	448	0.017	>430
Vitamin D (μg)	4.27	5.22	0.020	>4.7	3.96	4.42	0.013	>4
Vitamin E (mg)	7.59	8.51	0.003	>7.6	7.09	7.39	0.075	–
Vitamin B_12_ (μg)	4.78	5.77	0.002	>4.8	4.33	4.90	0.003	>4.4
Folic acid (μg)	289	323	0.001	>290	276	293	0.004	>280
Pantothenic acid (mg)	74.8	82.0	0.015	>6.5	80.0	86.4	0.100	–
Vitamin C (mg)	74.8	82.0	0.022	>75	80.0	86.4	0.014	>81
Cholesterol (mg)	398	412	0.252	–	328	354	0.007	>330
Insoluble dietary fiber (g)	11.5	12.9	0.001	>12	10.9	11.5	0.007	>11
*n*-3 fatty acids (g)	12.4	12.9	0.003	>2.3	1.95	2.04	0.353	–
Se (μg)	82.7	86.2	0.144	–	61.5	68.1	0.002	>62
Biotin (μg)	42.2	45.1	0.033	> 43	36.6	39.6	0.000	> 37

Values shown as means for each subgroup (low group: participants with a subjective accomplishment score < 7.0; high group: participants with a subjective accomplishment score ≥ 7.0). The cutoff refers to the recommended level of each nutrient to improve SA. Mann–Whitney U tests were used for comparison between subgroups. K: potassium; Mg: magnesium; P: phosphorus; Fe: iron; Cu: copper; Se: selenium.

**Table 3:** Nutrient content of the test meals.

Nutrients	Meal 1	Meal 2	Meal 3	Meal 4	Meal 5	Meal 6	Meal 7	Meal 8	Meal 9	Meal 10	Average
Total energy (kcal)	634.3	733.3	647.2	689.8	665.0	647.8	600.9	624.6	546.9	697.5	648.7
Protein (g)	35.6	28.8	34.9	29.0	25.6	32.0	32.7	32.6	15.2	28.7	29.5
Fat (g)	19.4	26.7	24.5	17.8	19.6	17.1	19.8	19.3	22.9	28.3	21.5
Carbohydrate (g)	88.1	102.8	77.8	111.4	104.7	98.3	83.6	87.7	76.1	91.6	92.2
Ash (g)	5.6	4.0	4.6	4.6	5.1	4.5	6.5	4.8	4.1	4.7	4.9
Na (mg)	825.4	518.4	671.9	685.0	813.5	785.6	858.2	700.6	519.7	732.3	711.1
** K (mg)	1240.7	901.8	1103.4	1063.3	1217.6	917.0	1702.2	1075.5	1049.6	964.8	1123.6
** Mg (mg)	115.3	113.1	102.6	102.7	99.6	99.3	145.5	99.1	105.1	128.6	111.1
** P (mg)	508.2	399.4	483.6	413.0	368.0	409.6	491.9	444.9	260.2	424.3	420.3
* Fe (mg)	4.3	3.6	3.8	3.0	3.9	2.4	3.4	3.7	4.3	4.2	3.7
* Cu (mg)	0.5	0.4	0.4	0.4	0.4	0.3	0.6	0.4	0.4	0.5	0.4
** Vitamin A (μg)	563.4	294.3	182.2	291.1	432.2	361.9	557.9	440.1	421.8	300.8	384.6
* Vitamin D (μg)	2.0	2.8	1.1	0.2	0.2	0.1	3.1	2.5	0.1	3.1	1.5
** Vitamin E (mg)	6.6	16.8	12.0	11.3	14.0	7.2	7.6	11.6	16.7	17.1	12.1
* Vitamin B_12_ (μg)	0.7	3.3	1.2	0.4	0.3	0.2	3.9	0.7	0.2	7.9	1.9
** Folic acid (μg)	185.3	201.8	130.9	119.9	149.5	104.7	183.9	156.6	214.5	154.6	160.2
* Pantothenic acid (mg)	3.7	2.0	2.1	2.1	2.2	2.7	2.4	2.4	1.1	1.7	2.2
** Vitamin C (mg)	74.1	83.4	50.8	46.5	68.9	68.0	27.0	97.1	64.1	53.7	63.4
Cholesterol (mg)	235.5	55.0	121.5	83.0	70.0	66.5	115.1	201.1	21.5	48.0	101.7
** Insoluable dietary fiber (g)	15.3	14.4	12.2	16.5	13.0	14.5	14.3	15.0	12.1	13.0	14.0
* *n*-3 fatty acids (g)	0.4	2.3	1.2	0.6	0.5	0.5	1.6	0.5	1.3	2.4	1.1
* Se (μg)	28.6	27.9	27.9	19.7	19.8	19.7	29.2	31.8	4.9	50.5	26.0
* Biotin (μg)	28.1	11.0	14.3	12.2	11.3	8.8	18.3	19.3	10.0	13.9	14.7

** Nutrients that reached one third of the recommended daily intake in every meal. * Nutrients that reached one third of the recommended daily intake on average over 10 meals. K: potassium; Mg: magnesium; P: phosphorus; Fe: iron; Cu: copper; Se: selenium.

**Table 4:** Characteristics of participants in the intervention study.

Age Groups	Male		Female	
	*n*	%	*n*	%
20–29	0	0.0	0	0.0
30–39	3	3.2	7	7.4
40–49	7	7.4	12	12.6
50–59	16	16.8	9	9.5
60–69	7	7.4	9	9.5
≥70	15	15.8	10	10.5
Total	48	–	47	–

The participants included had both meal and questionnaires records for at least one workday during the intervention period. n: number of participants. %: percentage among percentage among all participants.

## Data Availability

Data are contained within the article.
